# Hydrogen Bonding Network in Interlayer Spaces of a Partially Deuterated Layered α‐Sn (IV) Phosphate: A Solid‐State MAS NMR Study

**DOI:** 10.1002/mrc.70094

**Published:** 2026-03-05

**Authors:** Vladimir I. Bakhmutov, Hong‐Cai Zhou

**Affiliations:** ^1^ Department of Chemistry Texas A&M University College Station Texas USA

**Keywords:** ^1^H, ^2^H, and ^31^P MAS NMR data, deuteration, nuclear relaxation

## Abstract

Samples of a layered α‐Sn (IV) phosphate were partially deuterated by soaking with D_2_O to yield a mixture of two isotopomers Sn (HPO_4_) (DPO_4_).c‐H_2_O and Sn (DPO_4_)_2_.c‐H_2_O containing cavity water c‐H_2_O. They were characterized by the ^1^H, ^2^H, ^31^P, and ^119^Sn MAS NMR experiments including relaxation time measurements. The formation of these isotopomers is proven by the kinetic proton–deuterium cross‐polarization MAS NMR experiments giving the cross‐polarization rate constant *T*_H‐D_ of 3.2 ms. In agreement with their formulation the ^2^H MAS NMR spectra of Sn (HPO_4_) (DPO_4_).c‐H_2_O and Sn (DPO_4_)_2_.c‐H_2_O did not display the other signals besides the DPO_4_ resonance. The DPO_4_ groups observed in the temperature‐independent ^2^H MAS NMR spectra show the DQCC value of 184 ± 6 kHz corresponding to hydrogen bonds formed with an O^**…**^ O distance estimated as ~2.7 Å. Because of reduced dipolar interactions in the deuterated samples, the ^1^H MAS NMR spectra are well resolved providing signal assignments and the analysis. According to the solid‐state NMR data collected for the partially deuterated samples of **SnP**, the cavity water accepts one hydrogen bond from the P‐OH donor group and forms one hydrogen bond with the neighboring phosphate group, while the other water hydrogen is not involved in hydrogen bonding.

## Graphical abstract

The solid‐state ^1^H and ^2^H NMR data collected for deuterated α‐Sn (HPO_4_)_2_ phosphate suggest the hydrogen bonding network, where the cavity water accepts one hydrogen bond from the P‐OH donor group and forms one hydrogen bond with the neighboring phosphate group, while the other water hydrogen is not involved in hydrogen bonding.

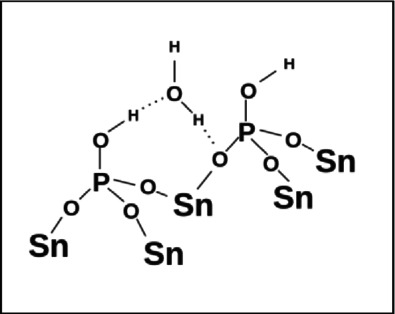

## 1 Introduction

Wide applications of tetravalent metal phosphate materials [1, 2, 3] in catalysis [4], proton conductivity [5, 6], and drug delivery [7] are conditioned by their layered structure and properties of acidic phosphate groups and water molecules in interlayer spaces. This structure is created by the stacking layers via either long hydrogen bonds or van der Waals forces [8] leading to the formation of cavities with a diameter of ~2.6 Å [7]. In general, the cavities are occupied by crystallization water [9] stabilizing the macrostructure of M (HPO_4_)_2_·H_2_O due to hydrogen bonds in intralayer and/or interlayer spaces. The present work is focused on the hydrogen bonding network in one of the important members of the layered materials, α‐Sn (IV) phosphate (**SnP**) [10] earlier investigated by x‐ray powder diffraction [11]. However, hydrogens of water and phosphate groups were not localized in this study.

The closest analog of **SnP**, a layered α‐Zr phosphate (**ZrP**), has been comprehensively studied by single crystal x‐ray method [12], neutron powder diffraction [13], and ab initio calculations [14, 15]. According to the studies, a water molecule in each cavity of the material accepts two hydrogen bonds from the P‐OH donor groups with O^…^ O distances of 2.807 (3) and 2.769 (3) Å [12]. In turn, the water acts as a donor forming one hydrogen bond with a P‐O‐H oxygen, while the other water hydrogen remains free. The latter is confirmed spectroscopically by the ^2^H MAS NMR spectra of a crystalline Zr (HPO_4_)_2_·D_2_O, where the cavity water displays two D_2_O resonances with *δ* (iso) of 8.5 ppm (H‐bonded hydrogen) and 3.7 ppm (free‐hydrogen) [16]. Nevertheless, according to the ab initio calculations [15], reorienting water molecules in **ZrP** easily leads to the structure, where both water hydrogens are free.

Potentially, the hydrogen bonding mode in compound **SnP** can be recognized by ^1^H solid‐state MAS NMR. However, the broad lines in the ^1^H MAS NMR spectra of **SnP** caused by strong proton–proton dipolar interactions [10] did not provide valuable information. In the present work, we analyze ^1^H MAS NMR spectra recorded for partially deuterated samples of **SnP**, which show significantly improved spectral resolution due to reduced proton–proton dipolar interactions [17]. Because the application of this approach to the solution of the formulated task turned out to be nontrivial, the data presented here can be useful for researchers applying solid‐state NMR and working in the field of material science.

## 2 Methods

### 2.1 Materials

The 0.9‐g samples of the initial α‐tin (IV) phosphate, **SnP**, prepared by Dr. A. Contreras‐Ramirez as described in [10], were poured in glass vials and soaked in ~5 mL of D_2_O for 1 h. Then, the water was decanted and the samples were placed into a convection lab oven at either 120°C or 160°C for ~60 min yielding dry white powders marked here as **SnPD1** and **SnPD2**, respectively.

### 2.2 Solid‐State NMR Measurements

Solid‐state MAS NMR data were collected with a Bruker Avance‐NEO solid‐state NMR spectrometer (400 MHz for ^1^H nuclei) equipped with a standard three‐channel 4‐mm MAS probe head. A standard solid‐echo pulse sequence with optimized echo delays of 0.000095 s and recycle time delays of 5 s was used to collect the ^2^H MAS NMR spectra. The ^1^H MAS NMR spectra were recorded with 90° rf‐pulses (2.5 μs) at recycle times of 10 s to provide full nuclear relaxation. Direct nuclear excitation was applied for the ^31^P{^1^H} and ^119^Sn{^1^H} MAS NMR experiments. The external references in the ^1^H, ^2^H, ^31^P, and ^119^Sn NMR spectra were TMS, benzene‐*d*_6_ (*δ* of 7.1 ppm), (NH_4_)_2_HPO_4_ (*δ* of 1.3 ppm), and SnO_2_ (taken as −600 ppm). The proton–deuterium cross‐polarization ^2^H MAS NMR experiments were carried out with ^1^H rf‐pulses of 2.5 μs and the contact times varying between 500 and 10,000 μs. The cross‐polarization kinetic data were treated with a standard fitting computer procedure. ^1^H and ^31^P T_1_ times were measured at room temperature by standard inversion‐recovery experiments. All the ^1^H MAS NMR spectra of the compounds were obtained after subtraction of the spectra recorded with amply rotors under the same conditions and with the same numbers of scans in each case. Deuterium quadrupolar coupling constant (DQCC) calculations were carried out by sideband analysis using a program inside the Bruker Top‐Spin software. The variable‐temperature ^1^H, ^2^H MAS NMR experiments utilized the temperature control unit integral to the spectrometer without temperature correction for spinning rate.

## 3 Results and Discussion

**FIGURE 1:**
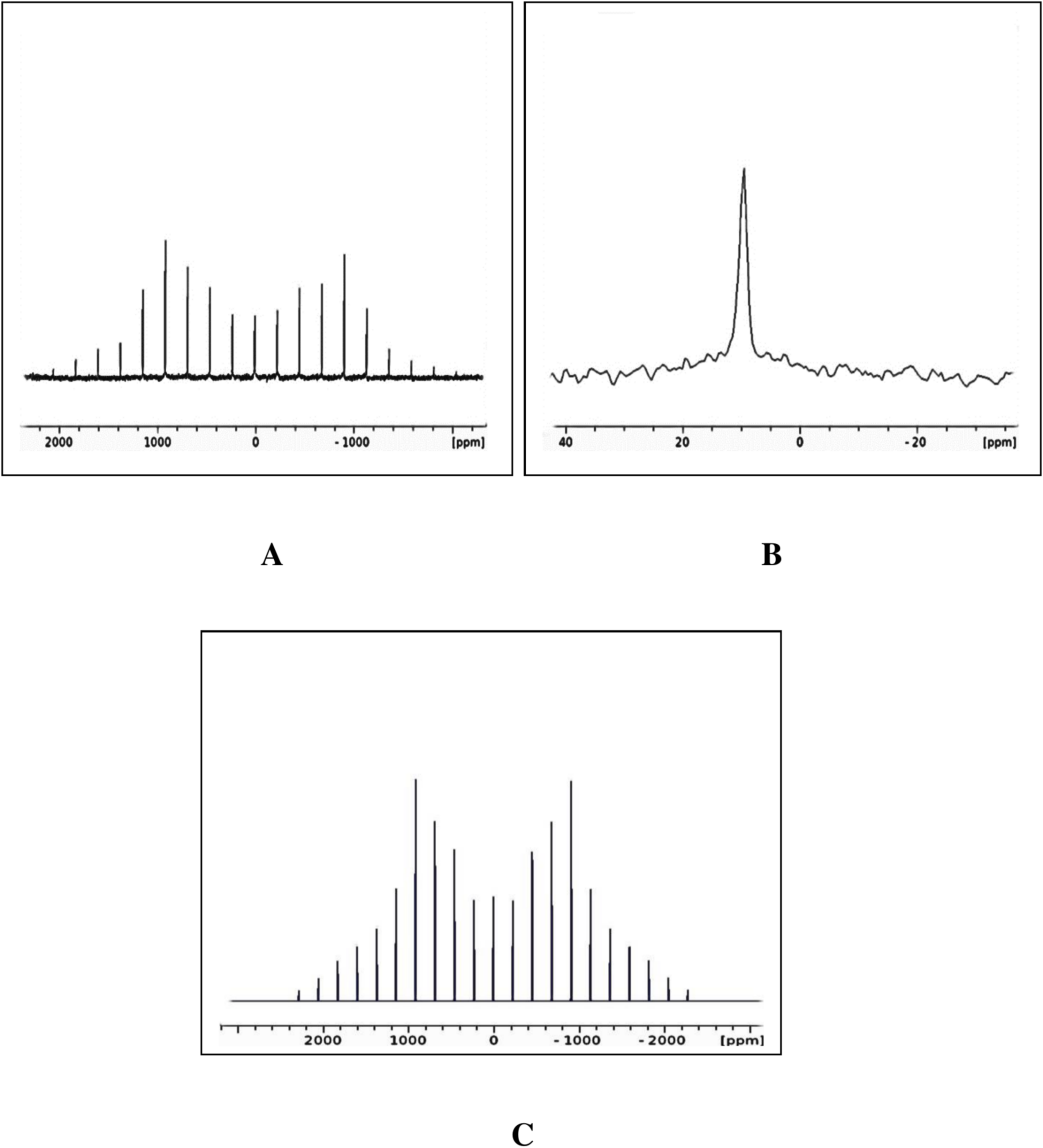
(A) The solid‐echo ^2^H MAS NMR spectrum of compound **SnPD2** recorded at 296 K and spinning rate of 14 kHz. (B) The isotropic signal in the same spectrum, respectively. (C) Theoretical ^2^H solid‐echo MAS NMR spectrum of **SnPD2** obtained for the DPO_4_ resonance.

**FIGURE 2:**
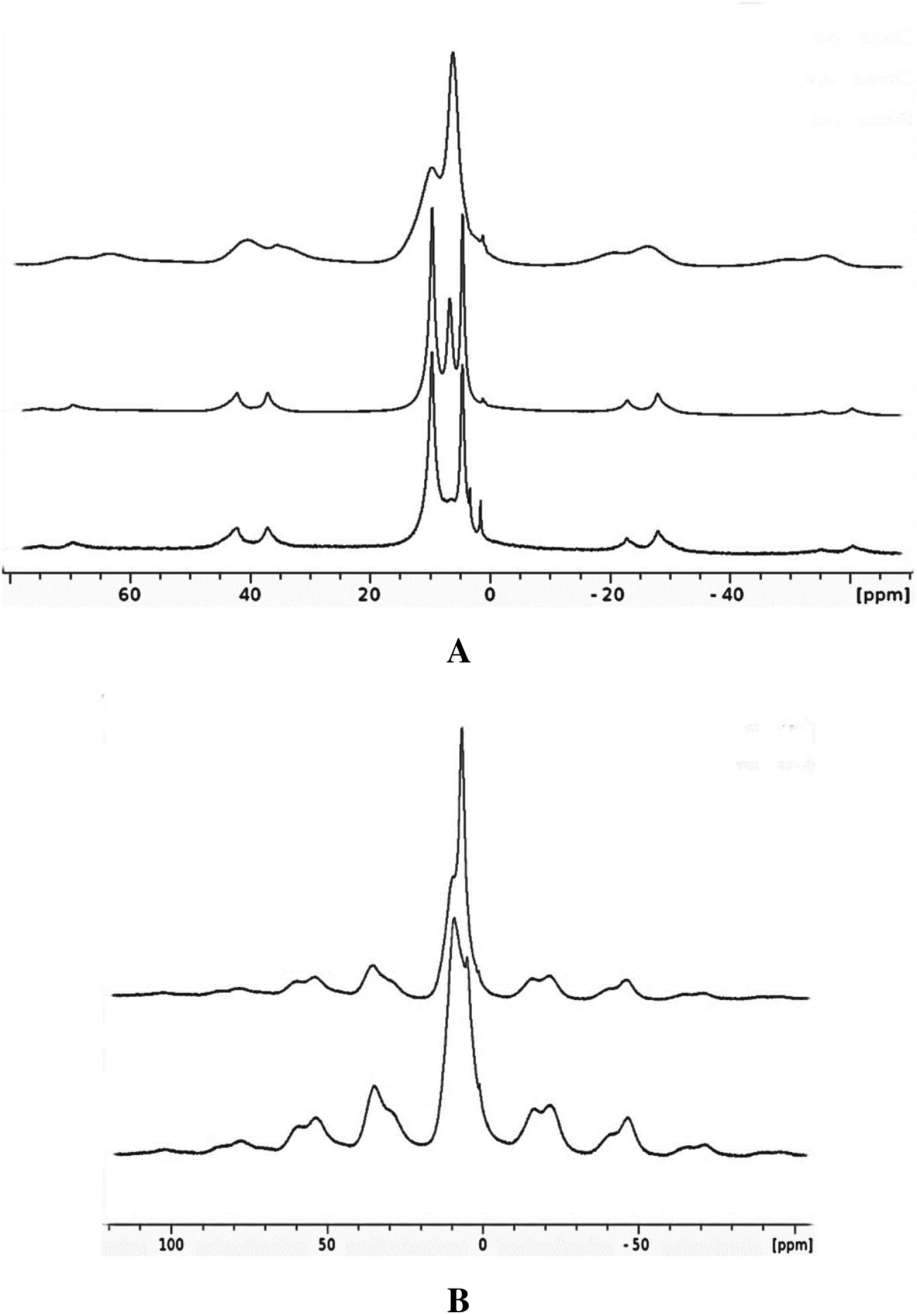
(A) Room‐temperature ^1^H MAS NMR spectra recorded at a spinning rate of 13 kHz from top to bottom: initial compound **SnP**, compound **SnPD1** and compound **SnPD2**. (B) ^1^H MAS NMR spectra recorded at a spinning rate of 10 kHz of initial compound **SnP** (top) and compound **SnP** dried at 160°C (bottom).

Percentage of the isotope exchange in samples **SnPD1** and **SnPD2** cannot be accurately determined by solid‐state NMR. Nevertheless, this exchange should be significant because the ^2^H MAS NMR spectra of deuterated samples represented by compound **SnPD2** in Figure 1 show the very intense and widely distributed quadrupolar pattern with a single and relatively sharp (Δ*ν* ≈60 Hz) isotropic resonance at *δ* (iso) of 9.6–9.8 ppm assigned to DPO_4_ groups on the basis of the ^1^HPO_4_ chemical shift in **SnP** [10]. Moreover, this deuterium quadrupolar pattern is well detected even after 64 scans with the intensity comparable to that observed in the ^31^P MAS NMR spectrum obtained under the same conditions (Figure S1) in spite of the strongly different phosphorus and deuterium gyromagnetic ratios and lower receptivity in the experiments on ^2^H nuclei. Finally, the strongest argument for a large content of deuterons in the lattice of **SnPD1** and **SnPD2** is the strongly improved resolution in their ^1^H MAS NMR spectra in Figure 2A providing rationalization of the collected data, signal assignments and their analysis.

As seen in Figure 2A, the initial nondeuterated compound **SnP** displays two broad overlapped proton resonances centered at *δ* (iso) of 9.6 and 6.1 ppm assigned earlier to hydrogen‐bonded HPO_4_ groups and water, respectively [10]. The signal at *δ* (iso) of 6.1 ppm reduces in sample **SnPD1** (dried at 120°C) and disappears in the ^1^H MAS NMR spectrum of **SnPD2** (dried at 160°C). Thus, this signal can now be assigned to water (marked as s‐H_2_O) situated on the surface of microcrystals [9]. Because the s‐H_2_O resonance in **SnP** and **SnPD1** is not accompanied by spinning sidebands (see below), the surface water is obviously highly mobile and shows a liquid‐like behavior. Besides the resonances at *δ* (iso) of 9.6 and 6.1 ppm, the ^1^H MAS NMR spectrum of **SnPD‐1** displays a sharp resonance at *δ* (iso) of 4.6 ppm (Figure 2A). Because this signal remains in the spectrum of sample **SnPD‐2** dried at 160°C, it can be attributed to water located in the cavities (c‐H_2_O). In fact, the cavity water can be removed from **SnP** only at temperatures ≥ 200°C [11]. On the other hand, such a signal could belong to SnO*H* sites forming due to a partial hydrolysis of **SnPD2**. However, this hypothesis can be ruled out because the ^119^Sn{^1^H} NMR MAS spectrum of **SnPD2** (Figure S2) displays a single resonance at *δ* (iso) of −805 ppm corresponding to Sn (HPO_4_) groups [10].

The resonance of c‐H_2_O in compounds **SnPD1** and **SnPD2** is high‐field shifted relative to surface water s‐H_2_O and its chemical shift, 4.6 ppm, is close to that of 3.7 ppm characterizing free hydrogen of cavity water in **ZrP** [16]. Therefore, there is no doubt that the high‐field signal of c‐H_2_O resonance in compounds **SnPD1** and **SnPD2 also** belongs to free hydrogens of the cavity water. As seen in Figure 2, the isotropic resonance at *δ* (iso) of 4.6 ppm (as well as the resonance at 9.6 ppm) in the ^1^H MAS NMR spectra of **SnPD1** and **SnPD2** is accompanied by the spinning sidebands caused by proton chemical shift anisotropy CSA [18] and dipolar interactions remaining unaveraged under MAS conditions. Thus, the behavior of the surface and the cavity water is different corresponding to their *different* ^1^H T_1_ times measured in compound **SnPD1** as 0.28 ± 0.02 and 0.72 ± 0.06 s, respectively. It is quite probable that the mobility of c‐H_2_O is restricted in contrast to s‐H_2_O. Finally, it should be added that the ^1^H MAS NMR spectra of **SnPD1** and **SnPD2** do not change with heating from 253 to 336 K excluding a proton–proton exchange between POH and c‐H_2_O at least on the NMR time scale.

To determine the hydrogen bonding mode of the cavity water investigated in the present work, two interpretational models can be considered focusing on the resonances at 9.6 and 4.6 ppm in the ^1^H MAS NMR spectra of **SnPD1** and particularly **SnPD2** (Figure 2A). In the absence of deuteration, the first model corresponds to a situation when the low‐field resonance (9.6 ppm) belongs to (HPO_4_)_2_ protons *only*, while the c‐H_2_O signal characterizes *two equivalent* free water hydrogens (note that this model does not agree with the neutron powder diffraction study of **ZrP** [13] but corresponds to the structure found by ab initio calculations [15]). Then, the integral intensities of the low‐ and high‐field signals should show a ratio of 1:1 (Sn (HPO_4_)_2_·H_2_O). In the second model, the c‐H_2_O water contains only one free hydrogen (4.6 ppm), while a bonded hydrogen signal is located closely to 9.6 ppm to yield the combined low‐field signal. In this case, the corresponding integrals should show a ratio of 3:1. Generally speaking, the second model is quite reasonable because firstly, linewidths of the resonances at 9.6 and 4.6 ppm in **SnPD2** (Figure 2A bottom) are different (Δ*ν* = 570 Hz vs. 430 Hz, respectively), and secondly, the hydrogen bonded water in **ZrP** displays the resonance in the low‐field region at *δ* (iso) of 8.5 ppm [16]. To support this model, we have carried out the ^1^H inversion‐τ‐recovery MAS NMR experiments on compound **SnPD2**. Figure S3 represents the spectrum obtained at τ of 0.1 s with FID treated with a Gauss function, where the low‐field resonance can actually be a superposition of two signals, one of which is sharp and similar to the signal at 4.6 ppm. This result can be considered as an argument, while the key moment for distinguishing the above models is the ratio between integrals of the corresponding signals.

As noted above, the resonances in the ^1^H MAS NMR spectra of nondeuterated compound **SnP** as well as a sample of **SnP** dried at 160°C are poorly resolved (Figure 2B) and their integration or deconvolution is impossible. In contrast, these manipulations can be carried out for the resonances in the well‐resolved ^1^H MAS NMR spectrum of **SnPD2** to yield a ratio of 1.51 ± 0.07 to 1. Following the first model, this ratio could illustrate an unreasonably increased content of HPO_4_ groups in spite of the deuteration. However, in terms of the second model the ratio of 1.51 is reasonable corresponding to the decreased content of ^1^HPO_4_ groups in a 50% mixture of two isotopomers Sn (HPO_4_) (DPO_4_).c‐H_2_O and Sn (DPO_4_)_2_.c‐H_2_O. This result implies a selective deuteration that is plausible because the ^2^H MAS NMR spectrum of **SnPD2** in Figure 1 does not display resonances of heavy water (HOD or D_2_O). It should be emphasized that even at coincidence of DPO_4_ and water resonances the ^2^H MAS NMR spectrum should show the second quadrupolar pattern because the DQCC are strongly different for these species [19]. It is remarkable that the D_2_O resonance appears in the ^2^H MAS NMR spectrum of **SnPD2**, when one drop of D_2_O was added directly into the rotor with the sample and shorty heated at 110°C. As shown in Figure S4, this resonance is Lorenz shaped corresponding to a liquid‐like behavior of s‐D_2_O at the absence of a deuterium–deuterium exchange with the DPO_4_ groups.

**FIGURE 3:**
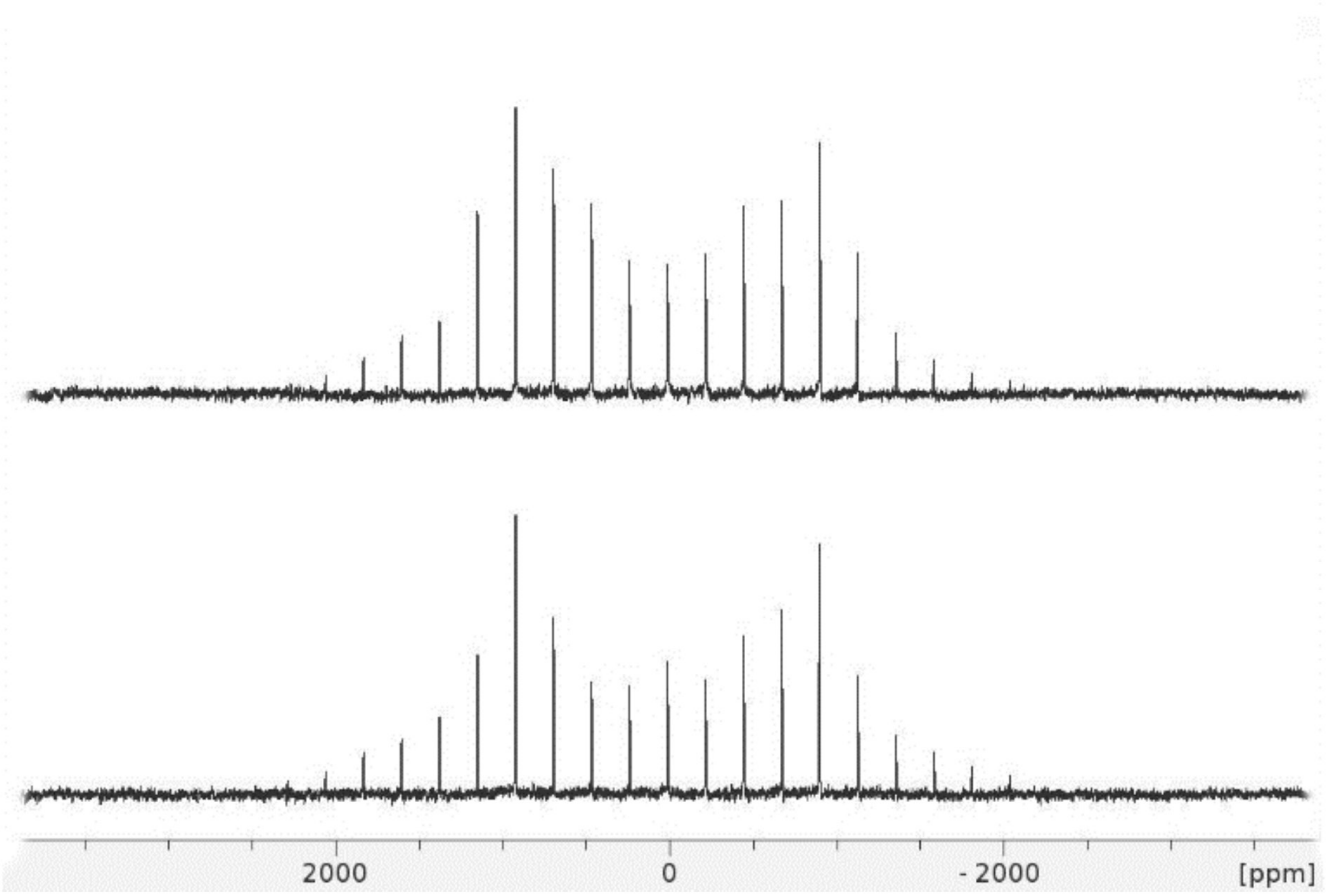
The solid‐echo ^2^H MAS NMR spectrum of compound **SnPD2** recorded at 296 K and a spinning rate of 14 kHz with 180 scans (top) and the ^1^H‐^2^H cross‐polarization MAS NMR spectrum of compound **SnPD2** recorded at a spinning rate of 14 kHz with 180 scans and a contact time of 6.0 ms (bottom).

To prove additionally the formation of isotopomers formulated as Sn (HPO_4_) (DPO_4_).c‐H_2_O and Sn (DPO_4_)_2_.c‐H_2_O we have carried out the ^2^H MAS NMR experiments on compound **SnPD2** applying proton–deuterium cross‐polarization [20, 21]. Figure 3 shows that this cross‐polarization is effective and results in the same quadrupolar pattern observed at direct nuclear excitation.

It is interesting that the cross‐polarization kinetics obtained by variations in contact times (*τ*) show a mono‐exponential behavior (Figure S5). The data fitted to equation *I*(*τ*) = *I*_0_[1 − exp(−*τ*/*T*_H‐D_)] [20] lead to the cross‐polarization rate constant *T*_H‐D_ of 3.2 ms, which is close to 2.5 ms reported for a polycrystalline 30% randomly deuterated hexamethylbenzene [21]. Thus, deuterons and protons in **SnPD2** are closely located with proton–deuterium distances similar to those in hexamethylbenzene. Finally, the presence of HPO_4_ groups in compound **SnPD2** is well supported by the ^31^P{^1^H} CP and ^31^P single pulse MAS NMR spectra in Figure S6, where they are compared with the spectra of initial compound **SnP**. As seen, firstly, the phosphorus resonances of **SnPD2** (HPO_4_ and DPO_4_) belonging to crystallographically inequivalent phosphate groups with *δ* (iso) of −12.3 and −13.3 ppm [10] show the effective cross polarization and, secondly, they are only slightly broadened in the single pulse ^31^P MAS NMR spectrum (Figure S6 bottom) due to unresolved ^2^J(^31^P‐^1^H) constants in HPO_4_ groups. In fact, this line broadening is reasonably much larger for initial nondeuterated compound **SnP**. It should be noted that the effect of deuteration in compound **SnPD2** on phosphorus relaxation could appear because of the weak dipole–dipole deuterium–phosphorus interactions in DPO_4_ groups. However, the ^31^P T_1_ time in **SnPD2** is very close to that in initial compound **SnP** and depends on spinning rates: 25.2 s at 5 kHz to 39.2 s at 10 kHz. This is in good agreement with the spin‐diffusion mechanism established for **SnP** [22], where the proton dipolar contribution is fully absent.

**FIGURE 4:**
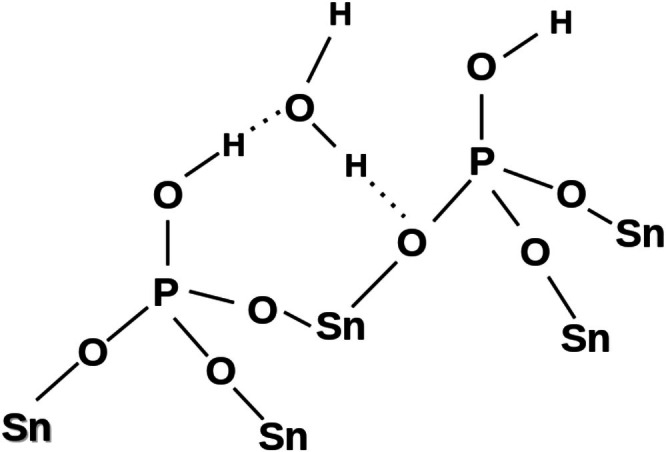
A structural fragment showing the hydrogen‐bonding mode of the cavity water in **SnP.**

Thus, summing the solid‐state NMR data discussed above for the partially deuterated samples of **SnP** leads to conclusion that one hydrogen of the cavity water forms a hydrogen bond with the neighboring phosphate group, while the other water hydrogen is not involved in hydrogen bonding. Schematically, this hydrogen bonding mode is shown in Figure 4 in agreement with the structure of **ZrP** represented in [14] and similar to that established for **ZrP** by solid‐state NMR [16].

The DPO_4_ groups of compounds **SnPD1** and **SnPD2** in the solid‐echo ^2^H MAS NMR spectra temperature‐independent between 253 and 336 K were characterized by simulations of the ^2^H quadrupolar patterns (Figure 1C) to give the DQCC value of 184 ± 6 kHz with an asymmetry parameter *η* of 0.08 corresponding to the axially symmetrical electric field gradients of OD groups [19]. This DQCC value is typical of acidic centers forming hydrogen bonds (Figure 4), which increase DQCC's correlating with the DQCC–HO^**…**^ O distances [19, 23]. As an example, phosphate KD_2_PO_4_ with short hydrogen bonds at an O^**…**^ O distance of 2.49 Å shows DQCC of 119.5 kHz [19]. Following this correlation, the DQCC value of 184 kHz will correspond to hydrogen bonds with an O^**…**^ O distance estimated as ~2.7 Å. Simialr distances, 2.807 (3) and 2.769 (3) Å, were found in the structure of **ZrP** [12, 13], where the cavity water accepts hydrogen bonds from the P‐OH donor groups.

The most disputable question in the present work remains a mechanism of the selective deuteration of acidic phosphate groups at soaking initial phosphate **SnP** with D_2_O. The formation of isotopomers Sn (HPO_4_) (DPO_4_).c‐H_2_O and Sn (DPO_4_)_2_.c‐H_2_O, itself, is well proved by the multi‐nuclear solid‐state MAS experiments, where deuterons of DPO_4_ are not exchangeable with protons of the cavity water. The absence of such an isotope exchange was already observed in the NMR study of the specially prepared crystalline‐layered zirconium phosphate Zr (HPO_4_)_2_.D_2_O [16]. In fact, in this case the cavity water was observed by ^2^H NMR MAS NMR and the phosphate groups appeared only in the ^1^H MAS NMR spectra. Add also that the HPO_4_/c‐H_2_O exchange is not visible on the NMR time scale in the variable‐temperature ^1^H MAS NMR spectra of compounds **SnPD1** and **SnPD2**, while the low‐frequency proton exchange between phosphate groups without participation of water molecules in **SnP** was established by ^1^H T_1ρ_ time measurements [10].

Generally speaking, such a selective deuteration of acidic P‐OH groups could be surprising or even implausible for homogeneous solutions. However, heterogeneous systems, like **SnP**/D_2_O, can have water‐inaccessible sites (WISs) [24]. In fact, liquid D_2_O must have access to sites potentially capable of isotope exchange in compound **SnP**, where the cavity spaces with a diameter of ~2.6 Å [7] are already occupied by the hydrogen‐bonded H_2_O. Therefore, in compound **SnP**, such WIS are acidic phosphate groups very active in ion‐exchanges with cation species [12, 25]. It is also difficult to rule out an uncontrollable reverse isotopic exchange of c‐D_2_O with H_2_O vapor at drying the samples [26]. Nevertheless, such a reverse exchange will be effective, first of all, for the surface water. Namely for this reason, the ^1^H MAS NMR spectrum of **SnPD1** (Figure 2A middle) shows the resonance of the surface water remaining invisible in its ^2^H MAS NMR spectrum. Thus, we believe that the WIS mechanism is most preferable to explain the selective deuteration. Finally, it should be emphasized that the above uncertainties do not affect the analysis and conclusions made in the present work.

## 4 Conclusions

A layered α‐Sn (IV) phosphate was treated by soaking with D_2_O to give a mixture of two isotopomers formulated as Sn (HPO_4_) (DPO_4_).c‐H_2_O and Sn (DPO_4_)_2_.c‐H_2_O and characterized by the ^1^H, ^2^H, ^31^P, and ^119^Sn MAS NMR experiments including relaxation time measurements. The formulation of these isotopomers is well confirmed by the kinetic experiments on the proton–deuterium cross‐polarization observed in the ^2^H MAS NMR spectra with variations in contact times giving the cross‐polarization rate constant *T*_H‐D_ of 3.2 ms. The solid‐echo ^2^H MAS NMR spectra of Sn (HPO_4_) (DPO_4_).c‐H_2_O and Sn (DPO_4_)_2_.c‐H_2_O recorded between 253 and 336 K have shown only one quadrupolar resonance belonging to DPO_4_ groups. They were characterized by the DQCC value of 184 ± 6 kHz corresponding to hydrogen bonds formed with an O^**…**^ O distance estimated as ~2.7 Å.

Because of reduced dipolar interactions, the variable‐temperature ^1^H MAS NMR spectra of the partially deuterated samples are well resolved, providing the signal assignments and their analysis. According to the ^1^H MAS NMR spectra, interlayer phosphate groups and cavity water are low mobile units in contrast to the high mobile and liquid‐like surface water. It is established that one of the cavity water hydrogens is free to show a high‐field ^1^H resonance at *δ* (iso) of 4.6 ppm.

Totality of the multinuclear solid‐state NMR data collected for the partially deuterated samples of **SnP** corresponds to the hydrogen bonding mode, where the cavity water forms one hydrogen bond from the P‐OH donor group and one hydrogen bond with the neighboring phosphate group, while the other water hydrogen remains free.

## Funding

This work was supported by the RDF grant of the Texas A&M University; US Department of Energy (DOE), Basic Energy Sciences (Grant Nos. DE‐SC0017864, DE‐SC0017864), and Welch Foundation (A‐0030).

## Conflicts of Interest

The authors declare no conflicts of interest.

## Supporting information


**Figure S1:** The ^31^P single pulse (top) and ^2^H solid‐echo (bottom) MAS NMR spectra of compound **SnPD2** recorded using the same number of scans (64), identical receiver gain values, and relaxation delays that provided full relaxation of the ^31^P (150 s) and ^2^H (5 s) nuclei.
**Figure S2:**: The ^119^Sn{^1^H} MAS NMR spectrum of **SnPD2** recorded using 50° rf‐pulses at a spinning of 12 kHz and relaxation delay of 15 s.
**Figure S3:** The isotropic parts of the ^1^H MAS NMR spectrum of **SnPD2** (top) and its ^1^H inversion‐τ‐recovery MAS NMR spectrum recorded at the τ value of 0.1 s when the intensity of H_2_O‐s was completely zero and the FID was treated with a Gauss function (bottom).
**Figure S4:**: The solid‐echo ^2^H MAS NMR spectrum recorded at a spinning rate of 10 kHz for a sample of **SnPD2**, when one drop of D_2_O was added directly into the NMR rotor containing the sample and shorty heated at 110°C.
**Figure S5:** Kinetics of the proton–deuterium cross‐polarization NMR MAS experiments (signal intensity vs. contact time) performed on a sample of **SnpD2**.The data are treated with a simple two spin model *I*(*τ*) = *I*_0_[*1* − exp(−*τ*/*T*_H‐D_)].
**Figure S6:** The one‐scan ^31^P MAS NMR spectra recorded at a spinning rate of 12 kHz from top to bottom: the ^31^P{^1^H} CP MAS NMR spectrum of **SnP** with a CP time of 2 ms; the single‐pulse ^31^P MAS NMR spectrum of **SnP**; the ^31^P{^1^H} CP MAS NMR spectrum of **SnPD2** with a CP time of 6 ms; the single‐pulse ^31^P MAS NMR spectrum of **SnPD2.**
