# Supplementary material for: Hydrogen Bonding Network in Interlayer Spaces of a Partially Deuterated Layered α‐Sn (IV) Phosphate: A Solid‐State MAS NMR Study
Source: Magn Reson Chem. 2026 Mar 5;64(5):521–7. doi: 10.1002/mrc.70094 (PMC13042208; doi:10.1002/mrc.70094)
Supplement: Supplementary file 1 — Figure S1: The 31P single pulse (top) and 2H solid‐echo (bottom) MAS NMR spectra of compound SnPD2 recorded using the same number of scans (64), identical receiver gain values, and relaxation delays that provided full relaxation of the 31P (150 s) and 2H (5 s) nuclei. Figure S2:: The 119Sn{1H} MAS NMR spectrum of SnPD2 recorded using 50° rf‐pulses at a spinning of 12 kHz and relaxation delay of 15 s. Figure S3: The isotropic parts of the 1H MAS NMR spectrum of SnPD2 (top) and its 1H inversion‐τ‐recovery MAS NMR spectrum recorded at the τ value of 0.1 s when the intensity of H2O‐s was completely zero and the FID was treated with a Gauss function (bottom). Figure S4:: The solid‐echo 2H MAS NMR spectrum recorded at a spinning rate of 10 kHz for a sample of SnPD2, when one drop of D2O was added directly into the NMR rotor containing the sample and shorty heated at 110°C. Figure S5: Kinetics of the proton–deuterium cross‐polarization NMR MAS experiments (signal intensity vs. contact time) performed on a sample of SnpD2.The data are treated with a simple two spin model I(τ) = I 0[1 − exp(−τ/T H‐D)]. Figure S6: The one‐scan 31P MAS NMR spectra recorded at a spinning rate of 12 kHz from top to bottom: the 31P{1H} CP MAS NMR spectrum of SnP with a CP time of 2 ms; the single‐pulse 31P MAS NMR spectrum of SnP; the 31P{1H} CP MAS NMR spectrum of SnPD2 with a CP time of 6 ms; the single‐pulse 31P MAS NMR spectrum of SnPD2. [file MRC-64-521-s001.docx]

**Hydrogen Bonding Network in Interlayer Spaces of a Partially-Deuterated Layered α‑Sn(IV) Phosphate: A Solid-State MAS NMR Study.**

Vladimir I. Bakhmutov* and Hong-Cai Zhou.

*Supplementary Material*


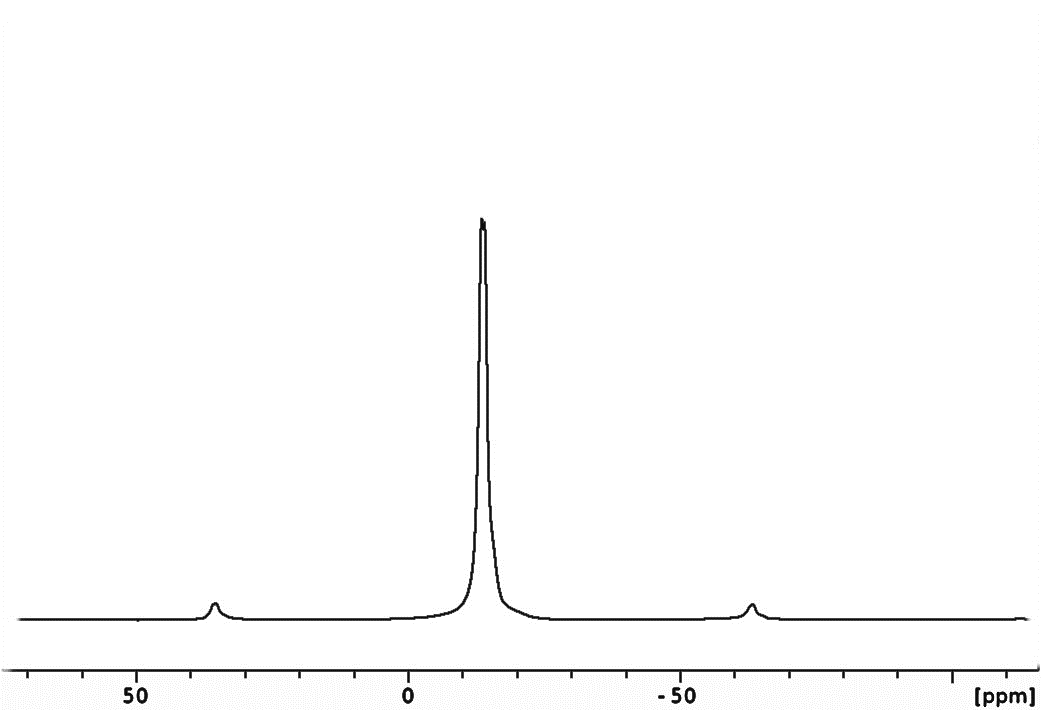


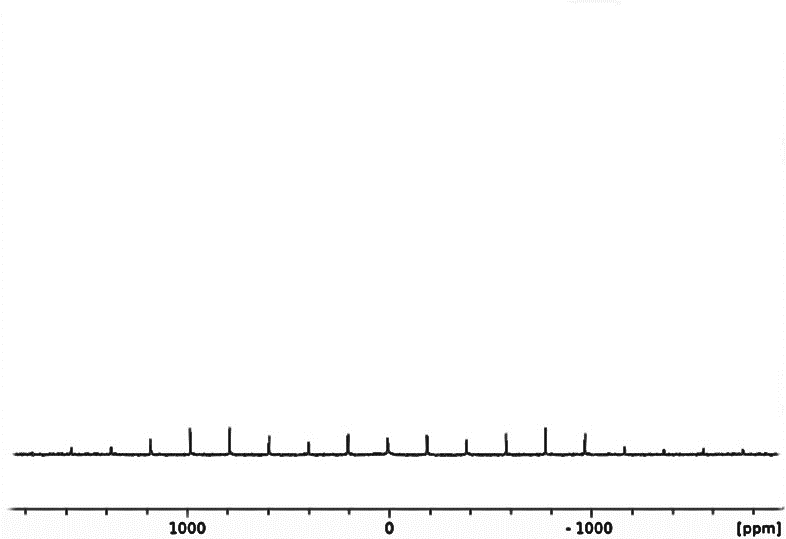


**FIGURE S1**. The ^31^P single pulse (top) and ^2^H solid-echo (bottom) MAS NMR spectra of compound **SnPD2** recorded using the same number of scans (64), identical receiver gain values, and relaxation delays that provided full relaxation of the ^31^P (150 s) and ^2^H (5 s) nuclei.


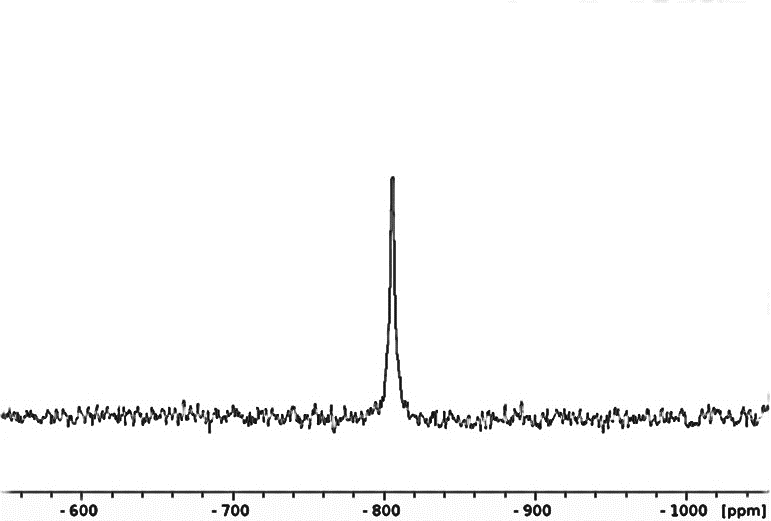


**FIGURE S2**. The ^119^Sn{^1^H} MAS NMR spectrum of **SnPD2** recorded using 50^o^rf-pulses at a spinning of 12 kHz and relaxation delay of 15 s.


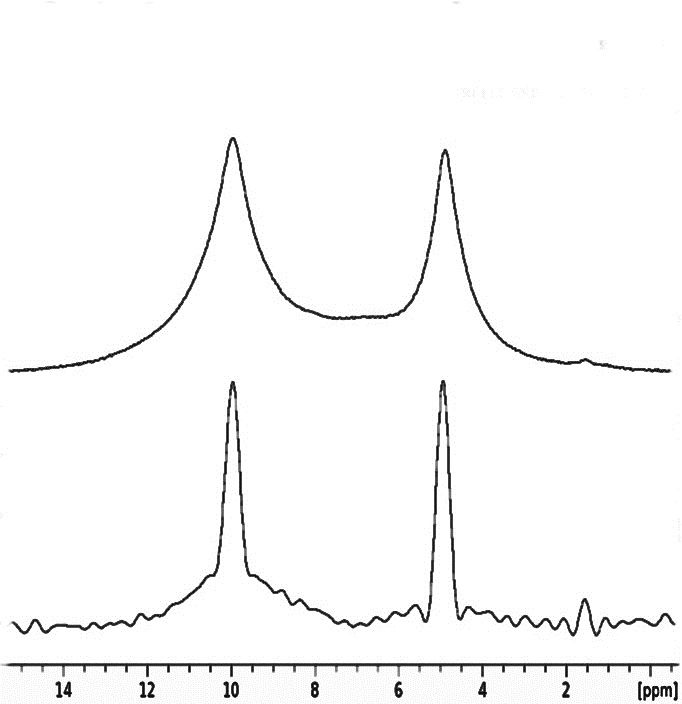


**FIGURE S3.** The isotropic parts of the ^1^H MAS NMR spectrum of **SnPD2** (top) and its ^1^H inversion-τ-recovery MAS NMR spectrum recorded at the τ value of 0.1 s when the intensity of H_2_O-s was completely zero and the FID was treated with a Gauss function (bottom).


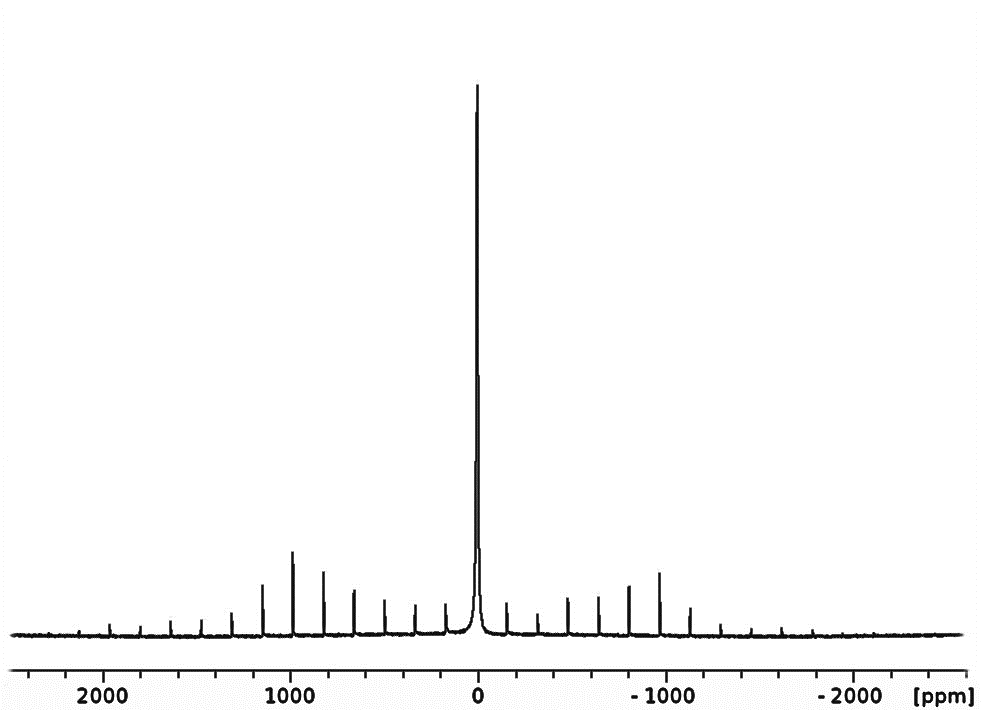


**FIGURE S4.** The solid-echo ^2^H MAS NMR spectrum recorded at a spinning rate of 10 kHz for a sample of **SnPD2**, when one drop of D_2_O was added directly into the NMR rotor containing the sample and shorty heated at 110^o^ C.

**I(au)**

**30**

**20**

**10**

**0**

**2500**

**CP time (μs)**

**7500**

**FIGURE S5.** Kinetics of the proton-deuterium cross-polarization NMR MAS experiments (signal intensity versus contact time) performed on a sample of **SnpD2**.The data are treated with a simple two spin model I(τ) = I_0_[1-exp(-τ/T_H-D_)].


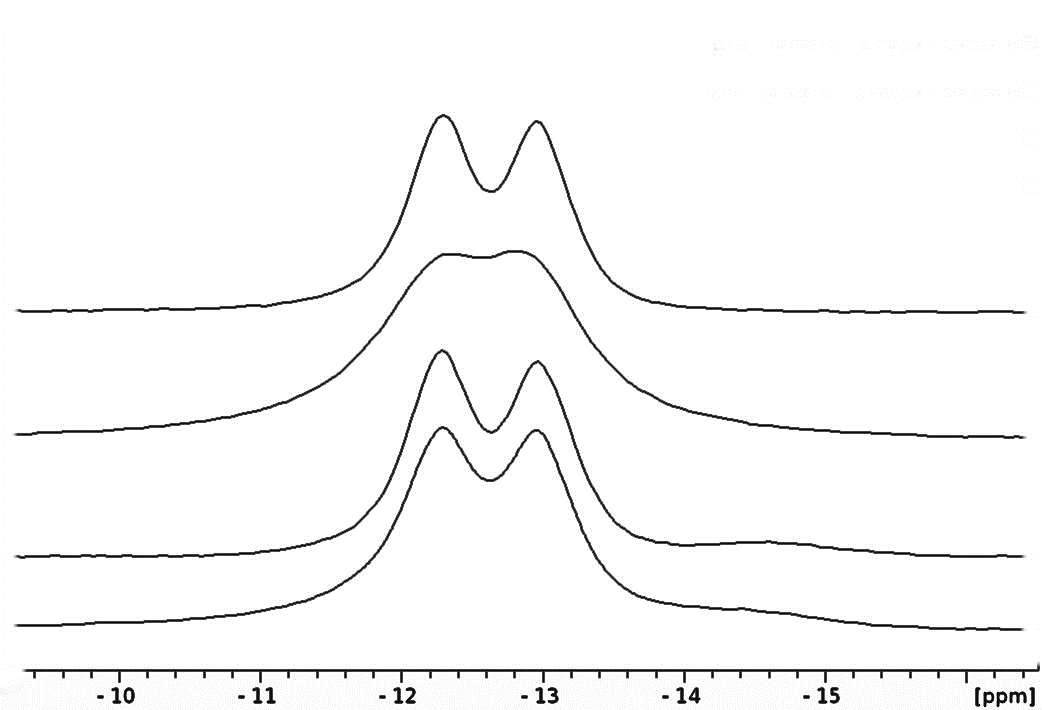


**FIGURE S6.** The one scan ^31^P MAS NMR spectra recorded at a spinning rate of 12 kHz from top to bottom: the ^31^P{^1^H} CP MAS NMR spectrum of **SnP** with a CP time of 2 ms; the single-pulse ^31^P MAS NMR spectrum of **SnP**; the ^31^P{^1^H} CP MAS NMR spectrum of **SnPD2** with a CP time of 6 ms; the single-pulse ^31^P MAS NMR spectrum of **SnPD2.**
